# Evaluating the scientific and regulatory case for the deployment of the radioresistant genus *Deinococcus* in bioremediation

**DOI:** 10.3389/fmicb.2026.1777693

**Published:** 2026-04-10

**Authors:** Sudeep S. Vattikuti, Leilani S. Boren, Ryan A. Grosso, Chad M. Albert, Jonathan T. E. Hill, Joaquin Caro-Astorga, James M. Tuohy

**Affiliations:** 1 Department of Biology, Glendale Community College, Glendale, AZ, United States; 2 School of Allied Health and Life Sciences, London South Bank University, London, United Kingdom; 3 Bioscience and Bioengineering Research Centre, London South Bank University, London, United Kingdom

**Keywords:** bioengineering, bioremediation, deinococcus, environment, extremophile, pollutants, radiation, regulation

## Abstract

Industrial activities and legacy contamination have generated metal-laden soils, radionuclide plumes, solvent-saturated sediments, and acidified pollutants. These are complex, hostile matrices where chemical treatments often redistribute rather than eliminate hazards, and where conventional mesophilic microbes cannot survive. Extremophiles, particularly species within the genus *Deinococcus*, represent a promising alternative for such environments. Their exceptional DNA repair systems and oxidative-stress resistance mechanisms enable metabolic activity under extreme conditions including ionizing radiation, prolonged desiccation, reactive oxygen species, and nutrient limitation. *Deinococcus* cells and biofilms adsorb metals through surface binding, and engineered strains can be designed to express redox pathways that convert soluble contaminants into insoluble, more readily recoverable forms. *Deinococcus* combines *in situ* applicability with minimal site preparation, exceptional stress resilience, and genetic adaptability, making it a strong candidate for bioremediation in environments resistant to conventional methods. This review explores the innate resilience of *Deinococcus*, its potential applications in bioremediation, and the prospects for enhancing its enzymatic repertoire through genetic engineering, culminating in a discussion of the challenges associated with scale-up and regulatory approval.

## 1 Introduction

The genus *Deinococcus* was first discovered in 1956 when Anderson et al. (1956) isolated an extraordinarily radiation-resistant bacterium from gamma-irradiated canned meat that had failed to remain sterile. Originally classified as *Micrococcus radiodurans* based on morphology, 16S rRNA phylogenetic analysis revealed sufficient distinctiveness to warrant reclassification into the new genus *Deinococcus* and family *Deinococcaceae* (Brooks and Murray, 1981). Since then, the genus has expanded to 93 validly published species (Göker et al., 2025) isolated from diverse extreme environments including desert soils, hot springs, Arctic regions, and radioactive sites (Rainey et al., 2005; Slade and Radman, 2011). Notably, the genus is free of any known human pathogens, distinguishing it from other commonly employed genera such as *Acinetobacter* and *Pseudomonas* (Goodfellow et al., 2012). Members of the genus, including *D. radiodurans*, have been detected at low abundance in gut microbiota of a range of organisms, including insects, birds, lizards, mammals, and multiple fish species, suggesting a broader ecological presence than previously appreciated (Chen et al., 2012; Ling et al., 2013; Hird et al., 2015; Miyake et al., 2015; Jiang et al., 2017; Egerton et al., 2018).

**Figure 1:**
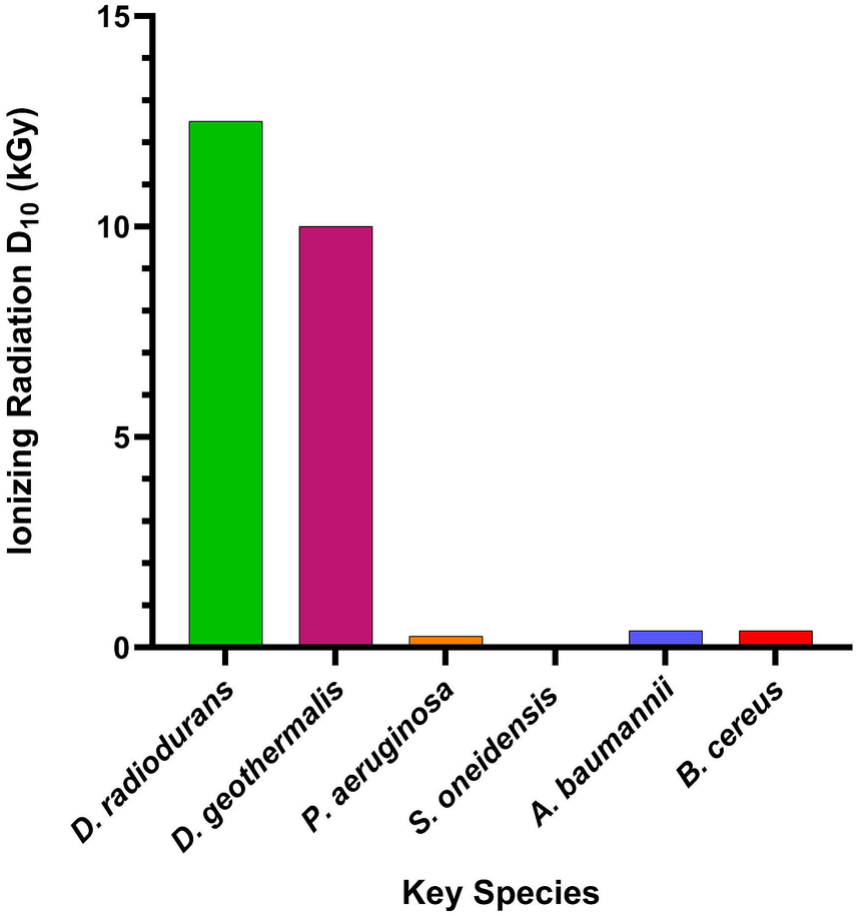
Radiation resistance of *Deinococcus* relative to other key species used in bioremediation. Compilation of D_10_ values for selected species: *D. radiodurans* (Daly et al., 2004), *D. geothermalis* (Daly et al., 2004), *Pseudomonas aeruginosa* (Ezzat, 2014), *Shewanella oneidensis* (Brown et al., 2015), *Acinetobacter baumannii* (Saha and Chopade, 2009), and *Bacillus cereus* (Kotiranta et al., 1999). Created using GraphPad Prism version 10.6.1 for Windows, GraphPad Software, Boston, Massachusetts USA, www.graphpad.com.

*Deinococcus* is a promising candidate organism for bioremediation in high-radiation mixed-waste environments where most bacterial species perform poorly (Figure 1). Other radioresilient organisms merit brief examination, both to define the landscape of possibilities and to underscore the distinctive advantages of *Deinococcus*. Among bacteria, *Kineococcus radiotolerans* SRS30216T is frequently cited for its *γ* radiation resistance (D_10_ ≈ 10–12 kGy), approaching that of *Deinococcus radiodurans* (Phillips et al., 2002). Among archaea, the hyperthermophile *Thermococcus gammatolerans* survives acute γ radiation doses of ≈ 7.5 kGy, (Jolivet et al., 2003; Tapias et al., 2009) underscoring that high dose tolerance is not unique to *Deinococcus*. Yet these alternatives are tightly constrained by habitat; *Kineococcus* is best characterized in specific contaminated industrial contexts, while *Thermococcus* is native to high temperature marine vents, environments far removed from the terrestrial, mixed waste sites where most remediation is needed. So, while it is true that there are species with comparable radiation tolerance, these species tend to have a high degree of habitat specificity and are not as well characterized as *Deinococcus*. In practice, radiation tolerance without ecological fit or tractable handling offers limited advantage.

In contrast, *Deinococcus* combines robust radioresilience (Lim et al., 2019) with exceptional ecological breadth and laboratory tractability. The genus has representatives adapted to a wide array of terrestrial and aquatic habitats across the globe. Desert and soil derived species include *D. deserti* (de Groot et al., 2005), *D. sonorensis* (Boren et al., 2024), *D. ficus* (Lai et al., 2006), and *D. arcticus* (Wang et al., 2019), while aquatic representatives include freshwater isolates such as *D. caeni* (Im et al., 2008) and *D. aquaticus* (Albert et al., 2023), as well as marine species like *D. rubrus* (Srinivasan et al., 2017) and *D. enclensis* (Thorat et al., 2015). Psychrophilic strains have been recovered from Antarctic dry valleys and alpine environments, including *D. saxicola* (Hirsch et al., 2004) and *D. altitudinis* (Callegan et al., 2008). At the opposite thermal extreme, moderate thermophiles such as *D. murrayi* and *D. geothermalis* (Ferreira et al., 1997) have been identified, although no species with an optimal growth temperature above 60 °C have been isolated to date. Finally, isolates from the upper atmosphere, such as *D. aetherius* (Yang et al., 2010) and *D. aerius* (Yang et al., 2009), highlight the genus’s extraordinary ecological breadth. *Deinococcu*s is not merely durable in principle but adaptable in practice.

Real-world applications for *Deinococcus* include leftover nuclear production and storage sites, such as the Savannah River [High-Level Radioactive Waste (HLW) Interpretation, n.d.] and the Hanford site (Hanford overview–Washington State Department of Ecology, n.d.), as well as uranium-contaminated wastewaters (Li et al., 2021) and deserted heavy metal mines (Giordani et al., 2024). Abandoned uranium mines affecting inhabitants on Navajo land, for example, have been marked for cleanup by the EPA and associated agencies (US EPA, R. 09, 2016). Weapons testing sites, like the Nevada Test site (Radioactive Waste Management, n.d.), present a complex contamination profile where low- and high-level chemical and physical pollutants coexist within environments dominated by persistent radioactive contamination. A comprehensive list of superfund sites is available and updated by the EPA (US EPA, O, 2015).

This review examines the innate advantages of *Deinococcus* in bioremediation, surveys the expanding toolkit for its genetic engineering, and concludes by outlining the key challenges that must still be addressed to realize its full potential.

## 2 Natural advantages of *Deinococcus*

Members of the genus *Deinococcus* are renowned for their extraordinary resilience to oxidative stress, heavy metals, and both ionizing and non-ionizing radiation (Lim et al., 2019). This section examines these capabilities, widespread across *Deinococcus,* which collectively position the genus as a compelling candidate for remediating contamination in extreme environments.

### 2.1 Robust DNA repair and radiation tolerance

*Deinococcus radiodurans* can survive and accurately reassemble its genome after sustaining hundreds of double-stranded breaks per cell following extreme ionizing radiation or desiccation (Minton, 1994; Zahradka et al., 2006; Cox et al., 2010). A key factor in the radiation resilience of *D. radiodurans* appears to be unusually high concentrations of intracellular manganese coupled with low iron, yielding a Mn/Fe ratio of approximately 0.24, compared to less than 0.01 in *E. coli* (Daly et al., 2004; Mota et al., 2024). Rather than DNA damage being the primary determinant of cell death, emerging evidence indicates that oxidative injury to the proteome dictates survival; preserving protein integrity is essential for the robust DNA repair processes characteristic of this organism (Krisko and Radman, 2013). Elevated intracellular Mn(II) levels in *D. radiodurans* drive the formation of low-molecular-weight antioxidant complexes with orthophosphates, carboxylates, amino acids, and short peptides. These complexes neutralize reactive oxygen species primarily through catalytic superoxide scavenging: Mn(II)-peptide complexes mimic superoxide dismutase (SOD) activity, converting superoxide radicals (O₂•^−^) to less reactive species without relying on enzymatic machinery (Peana et al., 2020). Orthophosphate plays a particularly critical role, interacting synergistically with Mn^2+^ and peptides to preserve the activity of large, multimeric enzymes, including essential DNA repair proteins, even under radiation doses sufficient to entirely obliterate DNA (Daly et al., 2010). These complexes selectively protect proteins, including key DNA repair enzymes, from oxidative inactivation, particularly protein carbonylation, while DNA, though chemically vulnerable, remains repairable when these enzymes are preserved (Culotta and Daly, 2013).

Beyond metal-ion homeostasis, *Deinococcus* deploys an extensive array of antioxidant defenses. These include highly expressed enzymes such as catalase, peroxidase, and superoxide dismutase, which collectively mitigate oxidative stress. Complementing these enzymatic systems are protective biomolecules like carotenoids, which further enhance resilience by quenching reactive oxygen species (Cox and Battista, 2005; Slade and Radman, 2011; Lim et al., 2019; Qi et al., 2020).

A broad repertoire of DNA repair pathways is utilized by *D. radiodurans*, including nucleotide excision repair, base excision repair, mismatch repair, and homologous recombination, together with specialized double-strand break repair processes such as single-strand annealing (SSA) and extended synthesis-dependent strand annealing (ESDSA; White et al., 1999; Makarova et al., 2001; Slade et al., 2009; Cox et al., 2010; Timmins and Moe, 2016; Sharma et al., 2024). Notably, *D. radiodurans* lacks the canonical SOS-regulated error-prone translesion synthesis (TLS) polymerases and the Ku/LigD-mediated non-homologous end joining (NHEJ) system described in certain other bacteria, showing consistency with its reliance on predominantly error-free repair mechanisms (Timmins and Moe, 2016). This absence of mutagenic repair pathways underpins its remarkable genetic stability, a trait of considerable value for engineered bioremediation strains (Lim et al., 2019; Zahradka et al., 2024). Comparative genomic surveys, however, reveal that NHEJ (Ku/LigD) and translesion synthesis genes are unevenly distributed across the *Deinococcus* genus: while some species possess Ku/LigD or specialized TLS polymerases, the reference *D. radiodurans* R1 genome lacks obvious homologs of these classical mutagenic systems (Lim et al., 2019; Sharda et al., 2020).

**Figure 2:**
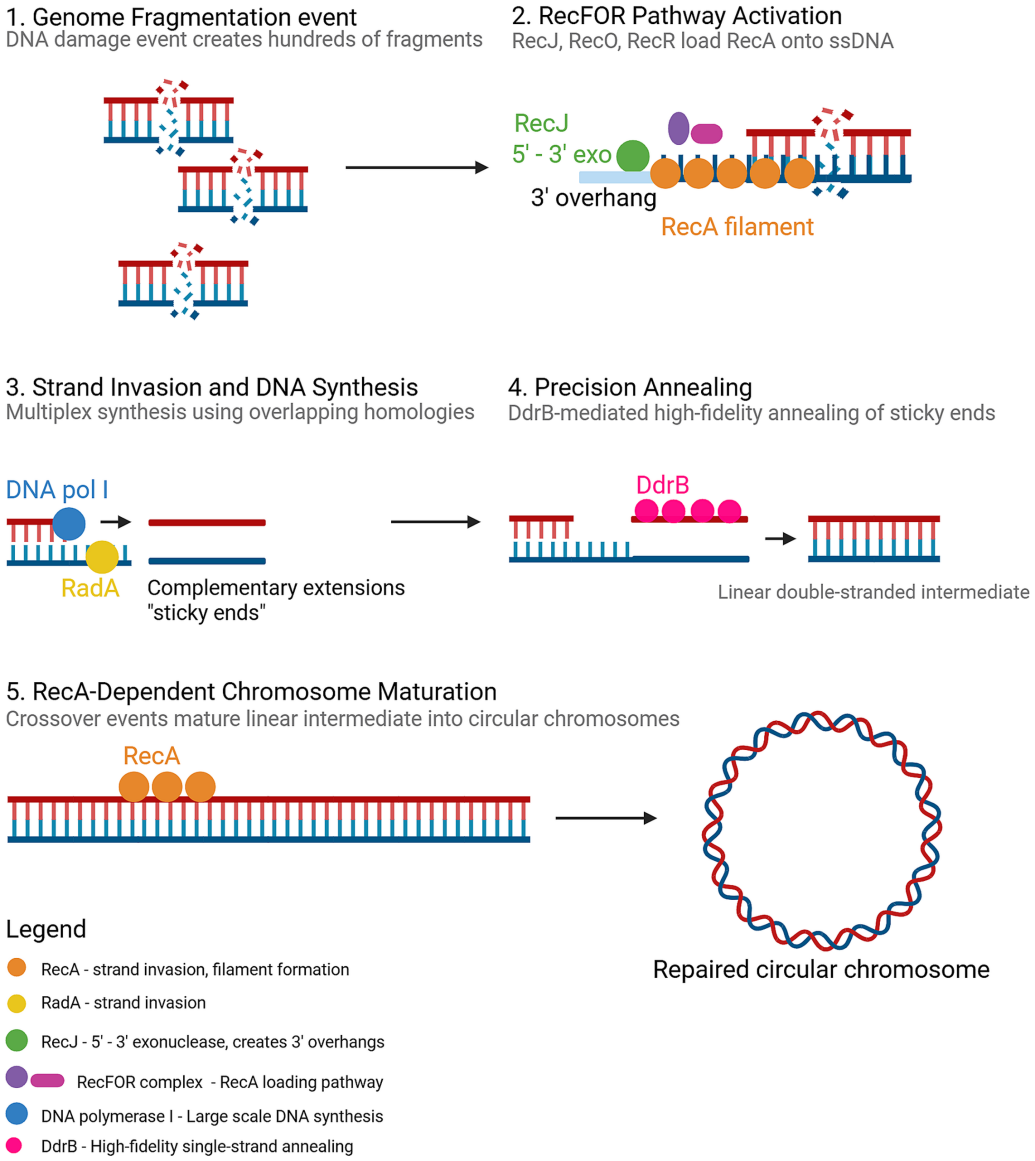
Extended synthesis-dependent strand annealing (ESDSA) in *Deinococcus radiodurans* R1. Created in BioRender. Albert (2026a) (https://BioRender.com/n9xti0i).

There are 4–10 genomic copies per cell in *D. radiodurans* (Hansen, 1978; Harsojo et al., 1981; White et al., 1999; Makarova et al., 2001), and these can provide overlapping homologous sequences to serve as primers and templates during large-scale DNA synthesis for genome reconstruction after irradiation. Following severe fragmentation, repair proceeds through extensive DNA synthesis from overlapping fragments and crossover recombination, which are processes that depend on polyploidy (Slade et al., 2009). Recovery occurs in two stages: an initial RecA-independent phase, where toroidal nucleoids and moderate polyploidy enable rapid end joining at short microhomologies to stabilize fragments (White et al., 1999; Englander et al., 2004), followed by ESDSA (Figure 2) and RecFOR/RecA-mediated homologous recombination. In this second phase, single-stranded fragments invade intact templates, DNA synthesis extends the invading strands, and overlapping tracts anneal to restore full chromosomes with high fidelity (Zahradka et al., 2006; Cox et al., 2010). Multiple genome equivalents are essential for ESDSA, increasing the likelihood of accurate homology search and copying, and underpinning the extraordinary genome reconstitution capacity of *D. radiodurans* (White et al., 1999; Makarova et al., 2001).

Under genotoxic stress in *D. radiodurans*, natural transformation (NT) proteins ComEA, ComEC, and EndA function primarily in metabolic rescue rather than direct genomic repair, processing extracellular DNA into nucleosides within the periplasm to sustain the cell’s repair machinery (Sharma et al., 2025). DSB repair proceeds through two temporally distinct pathways: an early RecA-independent SSA phase, followed by RecA-dependent ESDSA for genome reconstitution. DprA serves as a key regulator of this transition, modulating RecA loading onto single-stranded DNA through recombination mediator-like activity and competition with RecA loading factors. In Δ*dprA* mutants, repair shifts toward greater SSA reliance with reduced ESDSA involvement, indicating that DprA normally limits early SSA engagement while promoting transition to the later RecA-dependent pathway (Sharma et al., 2024). This positions DprA as a molecular switch governing both pathway selection and pathway timing, with RecA assembly representing the decision point between rapid initial repair and sustained genome reconstitution (Sharma et al., 2022b).

The PprI-DdrO regulatory circuit orchestrates the radiation response in many *Deinococcus* species (Lu and Hua, 2020; Lu et al., 2024). Under normal conditions, the transcriptional repressor DdrO binds Radiation/Desiccation Response Motifs (RDRMs), silencing at least 35 stress response genes (Devigne et al., 2015; Blanchard et al., 2017; Eugénie et al., 2021). This system is well characterized in species such as *D. deserti* VCD115 and *D. geothermalis* DSM 11300 (Ludanyi et al., 2014). Upon radiation exposure, the metalloprotease PprI cleaves DdrO at conserved sites (Wang et al., 2015; Qi et al., 2020), lifting repression and activating key DNA repair genes including *ddrA*, *ddrB*, *pprA*, *ssB* and *recA* (Selvam et al., 2013; Blanchard et al., 2017). This conserved proteolytic cascade enables rapid mobilization of repair systems against radiation damage (Daly, 2009; Blanchard et al., 2017).

In addition to transcriptional control by the PprI–DdrO system, *D. radiodurans* employs a post-translational regulatory pathway mediated by RqkA (DR2518), a eukaryotic-like serine/threonine protein kinase (STPK) induced by *γ*-irradiation. Since STPK-mediated regulation is relatively rare in bacteria, this highlights the unique regulatory complexity of *D. radiodurans*. Activated by pyrroloquinoline quinone (PQQ) following irradiation, RqkA phosphorylates multiple substrates central to DNA repair and cell cycle control (Rajpurohit and Misra, 2013; Sharma et al., 2022a; Misra and Rajpurohit, 2024). RqkA phosphorylates RecA at Tyr-77 and Thr-318, enhancing its DNA-binding and strand-exchange activities essential for post-irradiation recovery (Sharma et al., 2020; Rajpurohit et al., 2021). It also targets PprA at Thr-72, Ser-112, and Thr-144, increasing PprA’s DNA affinity and stimulating its end-joining function. Additionally, RqkA phosphorylates the cell division proteins FtsZ and FtsA in response to γ radiation, linking DNA damage detection to cell cycle arrest. Since *D. radiodurans* lacks the canonical LexA/RecA SOS system, this kinase-driven mechanism provides an alternative post-translational layer for coordinating DNA repair, genome stability, and cell division following extensive DNA damage (Rajpurohit and Misra, 2013; Rajpurohit et al., 2022; Misra and Rajpurohit, 2024).

### 2.2 Tolerance to oxidative stress

Although primarily recognized for its radiation resistance, *Deinococcus* also exhibits remarkable tolerance to oxidative stress. Reactive oxygen species (ROS), including free radicals such as superoxide and hydroxyl radicals, as well as non-radical species like hydrogen peroxide, can oxidize cellular components, disrupting metabolic functions and ultimately inducing cell death (Shields et al., 2021). Consequently, robust antioxidant defense systems are essential for survival in environments with elevated ROS levels, such as arid soils and heavy-metal-contaminated sites (Khan et al., 2024).

Hydrogen peroxide (H₂O₂) is a major oxidative threat, and *D. radiodurans* excels at neutralizing it, outperforming typical bacterial models. For example, *D. radiodurans* strains R1 and KD8301 exhibit EC₅₀ values for H₂O₂ scavenging of 0.12 mg/mL and 0.2 mg/mL of protein extract, respectively, whereas *Escherichia coli* requires a much higher concentration (EC₅₀ = 3.56 mg/mL) to achieve comparable effects (Tian et al., 2004). This ~30-fold difference highlights the potency of *Deinococcus* antioxidant systems, largely attributable to enzymatic defenses (catalases, superoxide dismutases, peroxidases) and nonenzymatic components such as Mn(II) complexes and carotenoids like deinoxanthin.

Beyond Mn(II)-mediated protection, *D. radiodurans* employs transcriptional mechanisms to fine-tune its oxidative stress response. Central to H₂O₂ sensing is a 1-Cys OxyR variant that functions as both a transcriptional activator and repressor (Chen et al., 2008). Upon oxidation by H₂O₂, OxyR activates *katE*, encoding a catalase that detoxifies H₂O₂ directly. Simultaneously, OxyR represses *dps* and *mntH*, genes involved in iron storage and manganese import, respectively. This repression limits free iron availability, thereby reducing Fenton-mediated hydroxyl radical production, while modulating manganese homeostasis to optimize the balance between enzymatic and non-enzymatic antioxidant systems. The PprI–PprM regulatory axis further contributes to oxidative stress management by controlling expression of additional protective proteins, including DR1127 and KatE1 (Lu et al., 2009; Li et al., 2017).

Adding a post-transcriptional layer, the small RNA PprS stabilizes PprM mRNA, thereby supporting expression of downstream DNA repair and oxidative stress genes (Villa et al., 2021). PprS knockdown impairs post-irradiation survival, underscoring its functional importance. This sRNA-mediated control complements transcriptional regulation by OxyR and PprI–DdrO and post-translational modulation by RqkA, completing a three-tiered regulatory architecture for stress response management (Rajpurohit et al., 2022).

Together, these systems efficiently neutralize ROS under extreme oxidative stress (Daly et al., 2004; Shashidhar et al., 2010; Slade and Radman, 2011; Qi et al., 2020). Within the genus, *D. proteolyticus*, *D. indicus*, and *D. grandis* exhibit antioxidant enzymatic activities comparable to *D. radiodurans*, whereas *D. ficus* and *D. mumbaiensis* display markedly lower catalase activity (Shashidhar et al., 2010). Consequently, careful species selection is essential for successful bioremediation in harsh environments.

### 2.3 Heavy-metal resistance and reduction

**Figure 3:**
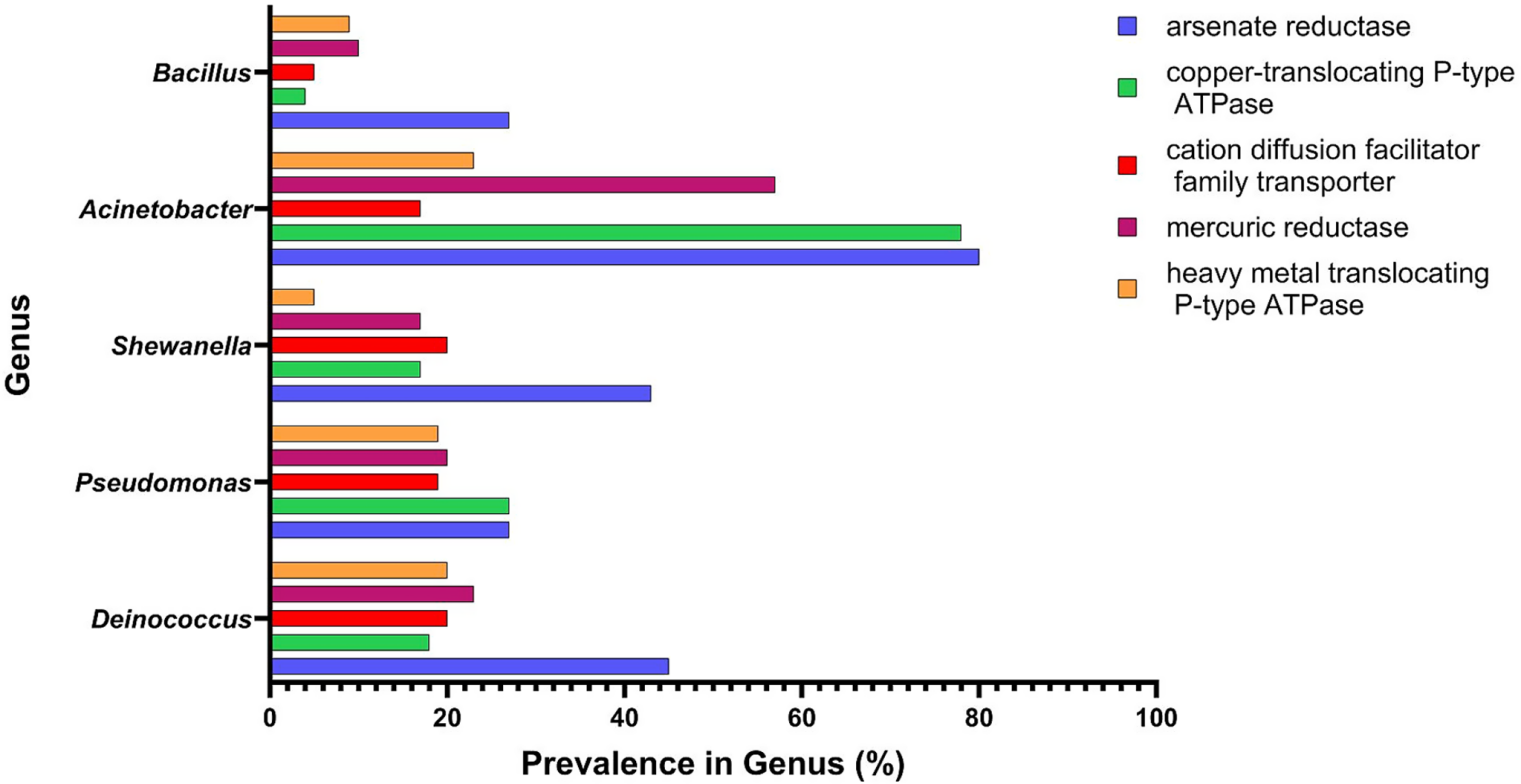
Proportion of species within each genus that possess representative heavy metal resistance genes. The comparison reflects genera commonly found in bioremediation. The results were compiled using the UniProt database (The UniProt Consortium et al., 2025). Created using GraphPad Prism version 10.6.1 for Windows, GraphPad Software, Boston, Massachusetts USA, www.graphpad.com.

Heavy metal contamination is common in abandoned mines, nuclear facilities, and polluted wastewaters. Effective bioremediation in these environments requires bacteria capable of metabolizing or detoxifying heavy metals. *Deinococcus* species combine exceptional radiation resilience with a high prevalence of metal-reduction genes, comparable to other leading bioremediation organisms (Figure 3).

Copper resistance in *D. radiodurans* is mediated by a copper-responsive gene cluster, comprising *copA, copZ,* and *csoR*. *CopA* functions as a P1-type ATPase that exports excess copper, *copZ* acts as a metallochaperone modulating copper distribution, and *csoR* serves as a copper-sensing repressor that derepresses the operon under elevated copper conditions (Zhao et al., 2014). Together, these proteins maintain copper homeostasis and bolster oxidative stress resilience.

Among *Deinococcus* species, *D. indicus* encodes copper-resistance genes alongside a mercuric-tolerance operon (Ranganathan et al., 2023). Its proteome has recently been mapped to reveal transporters for multiple metals, including chromium, cobalt, copper, iron, manganese, molybdenum, nickel, and zinc (Ramesh et al., 2025, preprint). When exposed to cadmium, *D. radiodurans* tolerates concentrations up to 5 mM (Khairnar et al., 2013), compared to 8 mM in the most resistant *Pseudomonas* and *Shewanella* strains (Sinha and Mukherjee, 2009; Raghad et al., 2016), and only 0.25 mM in wild-type *Bacillus subtilis* (Jiang et al., 2009).

Beyond copper and cadmium resistance, *D. indicus* is distinguished by extreme arsenic tolerance, harboring genes for three arsenate reductases (*diArsC*1–3). Remarkably, it grows in 1 M As(V), whereas *D. radiodurans* is inhibited at 200 mM (Gouveia et al., 2023), suggesting *D. indicus* may be the preferred host in arsenic-contaminated environments such as wastewater.

In addition, *D. radiodurans* reduces Fe(III)–nitrilotriacetic acid and Cr(VI), and in the presence of anthraquinone-2,6-disulfonate (AQDS), can also reduce U(VI), Tc(VII), and solid Fe(III) (Fredrickson et al., 2000). AQDS functions as an electron shuttle, facilitating these reductions. The bacterium, additionally absorbs up to 78% of cadmium and 86% of lead from 80 μM CdCl₂ and 200 μM PbCl₂ solutions, respectively (Dai et al., 2021), with efficiency further enhanced by genetic engineering (see Section 3.1, Bioengineering to Remediate Heavy Metals and Radionuclides). Following the cadmium and lead treatment, the total intracellular metal ion levels of wild-type cells were ~0.19 nmol/mg for Cd^2+^ and ~2.3 nmol/mg for Pb^2+^ ions.

Biofilm formation strengthens colony resilience and improves metal ion binding (Xing et al., 2022; Guo et al., 2023), while calcium treatment increases biofilm production and enhances remediation of nickel, cobalt, and uranium (Shukla and Subba Rao, 2015; Manobala et al., 2019, 2021; Guo et al., 2023). Mn supplementation has been shown to enhance biofilm formation in *D. radiodurans*, whereas Fe supplementation improves biofilm mass in *D. indicus*—findings valuable for improving metal reduction through biofilm expression (Gouveia et al., 2025). Future studies should investigate *Deinococcus* biofilms for biosorption of U(VI), Tc(VII), Fe(III), and Cr(VI), metals already reducible by planktonic cells.

### 2.4 Organic pollutants

Several strains of *D. radiodurans* can degrade diverse organic pollutants, including keratin and certain polycyclic aromatic hydrocarbons (PAHs). Dalmaso et al. (2015) showed that *D. radiodurans* produces extracellular metallopeptidases with keratinolytic activity, accompanied by sulfite release consistent with a sulfitolysis mechanism for breaking disulfide bonds. This capability supports bioremediation of keratin-rich waste such as feathers, hair, and horns.

Persistent organic pollutants (POPs), particularly PAHs, are of concern due to their toxic, mutagenic, and carcinogenic properties (El-Shahawi et al., 2010; Jackson et al., 2017). Bisht et al. (2010) isolated PAH-degrading bacteria from the rhizosphere of *Populus deltoides*, identifying *D. radiodurans* SBA6 as capable of utilizing both naphthalene and anthracene as sole carbon sources. After 6 days of incubation, SBA6 degraded 81.8% of anthracene and 27.8% of naphthalene in 50 mL minimal broth containing either 0.075 mM anthracene or 0.075 mM naphthalene respectively, highlighting its potential for PAH bioremediation.

## 3 Engineering *Deinococcus* strains for bioremediation

While *Deinococcus* exhibits broad natural reduction capabilities and exceptional radioresistance, (Chen et al., 1999; Dvořák et al., 2017), genetic engineering has further improved its efficiency and expanded its applicability for remediating mixed waste sites contaminated with radionuclides, heavy metals and organic pollutants.

### 3.1 Bioengineering to remediate heavy metals and radionuclides

Genetic engineering has significantly expanded the bioremediation potential of *Deinococcus* species, particularly *D. radiodurans* R1, for cobalt, uranium, mercury, cadmium, and lead. One of the earliest demonstrations of this was by Appukuttan et al. (2006), who introduced the *phoN* gene from *Salmonella enterica* into *D. radiodurans*. The expressed PhoN periplasmic acid phosphatase enabled efficient uranium bioprecipitation by forming insoluble metal phosphates on the cell surface. Radioresistance remained unchanged (D_10_ ≈ 18 kGy), while uranium precipitation exceeded 90% within 6 h in a 0.8 mM uranyl nitrate solution and stayed at 90% even after exposure to 6 kGy of ^60^Co *γ* radiation. Similarly, Jiang et al. (2024) engineered *D. radiodurans* to overexpress citrate synthase (Deino-Cs), achieving 91.6% uranium accumulation at optimal pH 5.0. Uranium binding occurred via biosorption to cell wall functional groups through complexation and chelation. Citrate overproduction chelated co-contaminating Al^3+^, preserving surface binding sites and enabling superior uranium removal under mixed-contaminant conditions typical of acidic, uranium-polluted soils.

Engineering efforts have also targeted radioactive cobalt (^60^Co), a significant concern due to its 5.27-year half-life and high-energy γ-emission. Gogada et al. (2015) expressed Ni/Co transporter genes (*nixA* from *Rhodopseudomonas palustris* and *nvoA* from *Novosphingobium aromaticivorans*) in *D. radiodurans*. Under optimized conditions (OD₆₀₀ of 1.0, pH 5.8, 90-min incubation, 8.5 nM initial Co), engineered strains removed over 60% of ^60^Co, achieving uptake of 12.0 μg/g dry weight biomass, outperforming equivalent *E. coli* transformants while retaining functionality up to 6.4 kGy irradiation. Importantly, remediation efficiency was maintained in the presence of Fe, Cr, and Ni, demonstrating selectivity under the mixed-contaminant conditions typical of nuclear waste sites.

Another strategy involves modifying the cell surface. Dai et al. (2021) showed that a *Δdr2577* mutant lacking the cell surface-layer absorbed cadmium and lead 2.2-fold and 3-fold more efficiently than the wild type. Beyond direct sequestration, *D. radiodurans* produced antioxidants that mitigated heavy-metal-induced oxidative stress in rice plants, highlighting potential agricultural applications.

Recombinant *D. radiodurans* has also been shown to remediate ionic mercury in aqueous DOE mixed waste sites. Toxic Hg (II) was reduced to elemental mercury (Hg (0)), using the mercuric ion reductase enzyme encoded by the *merA* gene from *E.coli* BL308, introduced by cloning the entire *mer* operon (Brim et al., 2000). Four expression vectors derived from pMD66 were tested with varying copies of *merA* inserted into the *D. radiodurans* genome, ranging from approximately 1 to 150 copies. There was a direct correlation between *merA* copy number and mercury reduction, as monitored spectrophotometrically. Importantly, gamma radiation resistance was maintained alongside exceptional metal resistance and reduction across all vectors, regardless of gene dosage. The addition of the *mer* operon together with the *tod* cassette, responsible for toluene metabolism, further enhanced the ability of *D. radiodurans* to remediate complex waste mixtures (Brim et al., 2000).

Due to the limited ability of *D. radiodurans* to grow at elevated temperatures, Brim et al. (2003) similarly bioengineered *D. geothermalis*, a species capable of growth up to 55 °C, by inserting a plasmid carrying the *mer* operon. Engineering a moderate thermophile, like *D. geothermalis*, is critical for remediating contaminated environments that remain warm due to radionuclide decay. This species also exhibited the ability to reduce Fe (III), U (VI), and Cr (VI), highlighting its potential for mixed waste remediation (Brim et al., 2003). Notably, *D. geothermalis* is known for forming thick biofilms, a trait that may further enhance its bioremediation capacity (Kolari et al., 2002).

As previously discussed, many species within the genus beyond *D. radiodurans* and *D. geothermalis* possess radioresistance and heavy metal remediation potential. Comprehensive profiling of these species and their capabilities could provide a broader range of options for future bioremediation of mixed waste sites.

### 3.2 Recombinant strains for remediating organic pollutants

Radioactively contaminated environments present complex bioremediation challenges that extend beyond radionuclide sequestration and heavy metal reduction. These sites are often co-contaminated with organic solvents, used in nuclear fuel processing and facility maintenance (Lange et al., 1998; OECD and Nuclear Energy Agency, 2005). Conventional microbes fail under such conditions because most organisms cannot withstand lethal radiation levels. In contrast, *Deinococcus* species, particularly *D. radiodurans* and *D. geothermalis*, combine exceptional radiation resistance with metabolic versatility, enabling simultaneous remediation of radionuclides and organic pollutants.

A landmark study by Lange et al. (1998) engineered *D. radiodurans* strain MD560 to express toluene dioxygenase (TDO) genes from *Pseudomonas putida* F1. The recombinant strain oxidized diverse organics, including toluene, chlorobenzene, 3,4-dichloro-1-butene, and indole, while retaining its high radiation tolerance. Notably, TDO activity persisted under continuous exposure to 60 Gy/h *γ* radiation, and the strain tolerated solvent concentrations exceeding those typically encountered at radioactive waste sites. This work established the feasibility of deploying engineered *Deinococcus* for mixed waste bioremediation involving both radionuclides and organic contaminants.

**Figure 4:**
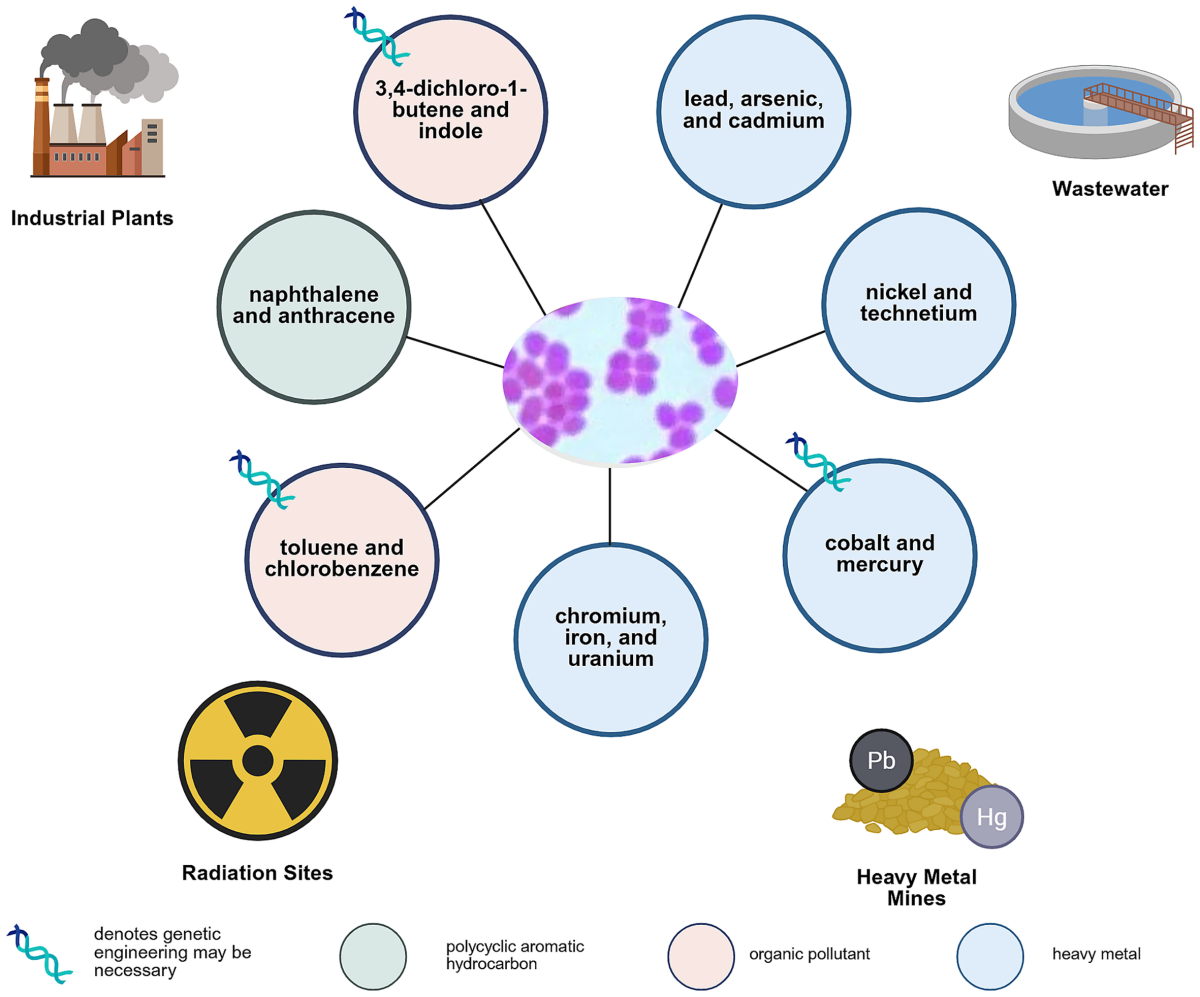
An overview of the pollutants that *Deinococcus* is capable of tolerating and/or bioremediating as well as the environments in which the organism could be deployed. Created in BioRender. Albert (2026b) (https://BioRender.com/loabcl7).

To date, four organic pollutants have been degraded by engineered *D. radiodurans*, expanding the repertoire of environments and compounds amenable to remediation (Figure 4). However, research on organic pollutant degradation in *Deinococcus* remains limited, likely due to the field’s emphasis on radiation resistance. This gap underscores the potential of leveraging *Deinococcus* not only for its resilience but also for its capacity to detoxify complex chemical mixtures in high-radiation environments.

## 4 Genetic engineering tools for *Deinococcus*

Advances in the bioremediation potential of *Deinococcus* have been driven by the rapid expansion of its genetic engineering toolkit. Conjugation protocols and shuttle vectors now enable efficient gene insertion, while systems such as SLICER, Cre-Lox, and CRISPR provide versatile options for targeted gene deletion. These tools underpin efforts to optimize metabolic pathways, incorporate biosafety “kill switches,” and enhance pollutant-degrading capacity. These are capabilities essential for field deployment in complex contaminated environments. The following section highlights key developments in these engineering strategies.

### 4.1 Gene delivery and expression

Genetic manipulation of *Deinococcus* species relies primarily on transformation and conjugation. Despite the structural challenges presented by a seven-layer cell envelope and a rigid, hexagonally packed S-layer (Liu et al., 2023; von Kügelgen et al., 2023), *D. radiodurans* has been transformable for nearly 60 years (Moseley and Setlow, 1968). Recent studies, notably Ithurbide et al. (2020) and Sharma et al. (2022a, 2022b), have defined the specific protein pathways required for DNA uptake. However, currently only a handful of Deinococcus species have been shown to be transformed (Table 1). This “transformation gap” may be attributable to a number of factors, including the potential lack of essential genetic machinery in non-model species and possibly a ‘species bias’, which serves to prioritize *D. radiodurans* over less-studied members of the genus.

**Table 1:** Major *Deinococcus* shuttle vectors, with plasmid size, transformation method, and selection marker.

*Deinococcus Species*	Shuttle vector	Plasmid size	Transformation method	Selection marker	References
*D. radiodurans*	pRAD1	6.3 kb	Ca*^a^*	Cmr*^e^*	Meima and Lidstrom (2000)
	pDEINO1	22.1 kb	Co*^b^*	Cmr	Brumwell et al. (2022)
	pSD	19.3 kb	Co	Neo*^f^*	Brumwell et al. (2023)
	pSLICER	16.2 kb	Co	Cmr	Brumwell et al. (2023)
*D. grandis*	pZT23	4.6 kb	E*^c^*, Ca	Cmr	Satoh et al. (2009)
	pGRC5	3.9 kb	Ca	Str*^g^*	Sakai et al. (2024)
	pRADN8	6.0 kb	Ca	Cmr	Sakai et al. (2024)
*D. deserti*	pI3	16 kb	E, Ca	Cmr	Dulermo et al. (2009)
	pBBR1MCS	4.7 kb	Co	Kan	Brutesco et al. (2017)
*D. geothermalis*	pMD66	28 kb	Ca	Tet*^h^*, Kan*^i^*	Brim et al. (2003)
*D. wulumuqiensis*	pK18mobsacB	7 kb	Na*^d^*	Kan	Chen et al. (2022)

*^a^* Ca, Calcium Chloride; *^b^* Co, Conjugation; *^c^* E, Electroporation; *^d^* Na, Sodium Chloride; *^e^* Cmr, Chloramphenicol; *^f^* Neo, Neomycin; *^g^* Str, Streptomycin; *^h^* Tet, Tetracycline; *^i^* Kan, Kanamycin.

Transformation has frequently been demonstrated in *D. grandis* and *D. geothermalis*. *D. grandis* may prove to be a robust bioremediation host. Its rod-shaped morphology provides a higher surface area to volume ratio than that possessed by its coccoid relatives, enhancing pollutant degradation rates (Harris and Theriot, 2018). *D. geothermalis*, a moderate thermophile capable of growth up to 55 °C, is well-suited for high temperature environments (Brim et al., 2003).

Recently, shuttle vectors pGRC5, pZT29H, and pRADN8 were introduced into *D. grandis*, establishing the first system capable of stably maintaining multiple foreign plasmids (Sakai et al., 2024). This advance is critical for bioremediation, as complex pathways are often best regulated on separate plasmids to avoid the instability that arises from oversized constructs (Sahota and Sharma, 2022). By distributing constructs across multiple vectors, researchers can avoid the instability that arises when oversized plasmids carry numerous genes. Comparable strategies have already proven effective in *Rhodopseudomonas palustris*, where dual plasmids enabled expression of an engineered mercury transport system for wastewater detoxification (Deng and Jia, 2011). Extending this approach by editing these new *D. grandis* vectors to encode waste-degrading enzymes could provide a powerful platform for environmental cleanup.

Brumwell et al. (2022) were the first to demonstrate bacterial conjugation in *D. radiodurans*, successfully transferring the 22 kb megaplasmid pDEINO1 from *E. coli*. Conventional calcium chloride transformation failed with this plasmid, highlighting the distinct advantage of conjugation for delivering large constructs. This capability is vital for bioremediation, as pathways such as the *mer* and *ars* clusters involve multiple sensor, chaperone, and transporter proteins (Boyd and Barkay, 2012; Mondal et al., 2021). Stable coexistence of vectors and retention of large DNA inserts therefore hold significant value.

Gene delivery in Deinococcus primarily relies on transformation, conjugation, and the expanding toolkit of shuttle vectors (Table 1). Future work should focus on engineering strains with pollutant-degrading pathways, as demonstrated in other microbes (Bang et al., 2000; Patel et al., 2010; Zhang et al., 2016), in order to fully unlock the bioremediation potential of the genus.

### 4.2 Promoters and reporter systems

#### 4.2.1 Constitutive promoters

In *Deinococcus*, the P*groES* promoter has been the most widely used due to its strong, constitutive activity (Meima et al., 2001). However, alternative promoters such as the weak *lexA* and moderately strong *amyE* have also been characterized in *D. radiodurans*, offering options for researchers seeking to minimize metabolic stress (Meima et al., 2001). This balance is critical in bioremediation, where excessive expression can burden host cells and compromise their survival, while weaker promoters may sustain long-term pollutant degradation (Bienick et al., 2014). Beyond these, novel promoters including *PDR_1261* and P*rpmB* have demonstrated even stronger activity than P*groES*, which may be more valuable for rapid detoxification of waste sites (Chen et al., 2019). Most recently, a library of 36 constitutive promoters spanning a 45-fold dynamic range was established in *D. radiodurans* and validated using the compact plasmid pAT00.20.001-sfGFP, which is smaller than pRAD1 (Simmons et al., 2025). These findings highlight a promising expansion of the genetic toolkit for tailoring expression strength to bioremediation needs—from high-level enzyme production to stress-tolerant, low-burden expression.

#### 4.2.2 Inducible promoters

Inducible regulation of catabolic pathways is essential for efficient bioremediation, as it minimizes metabolic burden while enabling bacteria to degrade pollutants only when needed (Santero and Díaz, 2020). Early work by Lecointe et al. (2004) adapted the Spac system to control gene expression in *D. radiodurans*. This system combines Pspac, a synthetic hybrid promoter derived from the *B. subtilis* spoVG promoter and the *E. coli* lac operator, with the lacI repressor, enabling precise and tightly regulated expression of essential genes upon IPTG induction.

Radiation responsive promoters have been identified in *Deinococcus* species. Luciferase assays have revealed two *pprA* promoters inducible by irradiation (Ohba et al., 2005). The *Pssb* promoter is part of the radiation/desiccation response (RDR) regulon and is strongly upregulated following DNA damage. Its use enhanced uranium removal more effectively than the constitutive P*groESL* promoter (Misra et al., 2014). In *D. grandis*, the *ddrO* promoter has been shown to respond to UV stress, reflecting the conserved nature of the RDR regulon across the genus (Devigne et al., 2015; Sakai et al., 2024).

Most recently, Simmons et al. (2025) reported that four small-molecule inducible systems in *D. radiodurans*, regulated by IPTG, salicylic acid, cuminic acid, and anhydrotetracycline, provided rapid and reversible control of gene expression.

Despite these advances, inducible promoters have been tested mainly in *D. radiodurans* and *D. grandis*, with limited application in other species. Moreover, most studies have focused on proof-of-concept rather than integrating inducible systems with pollutant degrading genes, leaving substantial opportunities for future bioremediation research.

#### 4.2.3 Reporter systems

Reporter systems validate gene expression by providing easily detectable outputs like light or color. Established options include *phoN* and *lacZ*. In the *phoN* system, cultures are supplied with p-nitrophenol phosphate, which is enzymatically converted into the yellow compound p-nitrophenol (Ujaoney et al., 2010). The amount produced directly reflects *phoN* activity and, by extension, plasmid expression levels. Similarly, *lacZ* encodes *β*-galactosidase, which hydrolyzes the substrate X-Gal to yield a distinct blue color, allowing straightforward measurement of reporter activity (Juers et al., 2012).

Light-emitting reporters have also been utilized for promoter activity assays in *Deinococcus* due to their sensitivity and ease of detection. The *lux* operon has been shown to produce functional luciferase in *D. deserti* (Brutesco et al., 2017), while firefly luciferase (*luc*+) has been used to monitor radiation-induced promoter activity in *D. radiodurans* (Ohba et al., 2005). In *D. grandis*, both deep-sea shrimp luciferase (*Nluc*) and firefly luciferase (*luc*+) were combined on a single plasmid and assayed with the Nano-Glo Dual-Luciferase system to analyze *ddrO* promoter activity (Sakai et al., 2024). GFP, widely used in *E. coli*, has likewise proven effective in *Deinococcus* (Narasimha and Basu, 2021).

Together, enzymatic, luminescent, and fluorescent reporters provide a versatile toolkit for tracking promoter strength and for monitoring engineered pathways in bioremediation.

### 4.3 Markerless genome editing

Markerless genome editing introduces genetic modifications without leaving behind selectable markers such as antibiotic resistance genes. This approach offers two major advantages; it allows multiple mutations to be introduced without marker interference (Kristich et al., 2005), and it minimizes the risk of horizontal gene transfer that could spread resistance traits to native microbial populations (Aminov, 2011). Such safeguards are particularly important in bioremediation, where engineered *Deinococcus* strains may be deployed at high density in environments containing other microbes. To date, homologous recombination remains the primary markerless editing strategy demonstrated in the genus, providing a foundation for developing safe and versatile bioremediation strains.

#### 4.3.1 Homologous recombination

Homologous recombination enables precise genome editing by integrating DNA constructs flanked with homologous sequences into the bacterial chromosome. In *D. radiodurans*, Brumwell et al. (2023) implemented a markerless knockout system using a seamless deletion cassette containing homology arms, an I-SceI recognition site, and selectable markers (*nptII* and *lacZ*). Following integration, bacterial conjugation was used to insert the helper plasmid (pSLICER), which expressed a codon-optimized I-SceI endonuclease (drI-SceI). Endonuclease-mediated cleavage induced recombination that excised both markers, and the plasmid was subsequently cured on non-selective media (Brumwell et al., 2023), yielding a scarless deletion platform for *D. radiodurans*.

In *D. geothermalis*, homologous recombination was used to evaluate 27 efflux pump genes for chromosomal integration (Boulant et al., 2021). The multidrug transporter MdfA exhibited the highest efficiency (Boulant et al., 2021). While this study focused on tolerance in microbial cell factories, efflux pumps are directly relevant to bioremediation; they export toxic metals such as silver, copper, and zinc, enhancing microbial survival in contaminated mining environments (Nies, 2003; Li and Ge, 2022; Gaurav et al., 2023).

The combination of polyploidy, superior recombination abilities and abundant repeat elements found in *D. radiodurans* dictates that the attainment of genetic homogeneity after genetic engineering requires extended selection. Reversion to wild-type has been well-documented when selective pressure is removed (Brumwell et al., 2022: Slade and Radman, 2011). However, in the context of bioremediation, this instability may be operationally advantageous. As environmental contaminant levels decline and selective pressure diminishes, reversion would reduce the persistence of engineered traits in the environment, a desirable outcome from a biosafety perspective.

#### 4.3.2 Cre-lox system

The Cre-Lox system provides a markerless genetic exchange pathway mediated by Cre recombinase, a tyrosine recombinase that recognizes paired *loxP* sites and excises the intervening DNA segment (Kim et al., 2018). In *D. radiodurans*, Jeong et al. (2017) established the first Cre Lox knockout system using two plasmids: pAM1, which carried loxP sites flanking a kanamycin resistance marker, and pAM2, which expressed Cre recombinase. Following homologous recombination, the *ddrO* gene was disrupted by double crossover, and subsequent Cre-mediated excision removed the kanamycin marker. The pAM2 plasmid was then cured by shifting the culture temperature from 30 °C to 37 °C, yielding a scarless deletion and demonstrating the feasibility of multiple gene knockouts in *D. radiodurans*.

This system enables temporal control of gene deletion, allowing insertion of a gene of interest followed by inducible Cre activity to remove selectable markers or target genes. Such control is particularly relevant to bioremediation, where metabolic resources must be conserved and pollutant degrading pathways may only be needed transiently. For example, polychlorinated biphenyls (PCBs) are degraded stepwise into chlorobenzoic acids and lighter congeners (Jing et al., 2018; Hassan et al., 2023), suggesting inducible or staged deletions could optimize resource use during complex degradation processes.

Together with the pSLICER system, Cre-Lox expands the Deinococcus genetic toolkit for bioremediation. Strategic deletions can eliminate counterproductive pathways, streamline metabolism, and support biosafety designs such as kill switches. Comparable work in *Pseudomonas putida* achieved eleven genomic deletions—including transposons and flagellar machinery—resulting in enhanced growth, stress tolerance, and improved replication of exogenous DNA (Martínez-García et al., 2014). These examples highlight the potential of markerless editing systems to strengthen a microbial chassis for environmental applications.

### 4.4 CRISPR-based modifications

A CRISPR-Cas12a (formerly known as Cpf1) system has recently been engineered in *D. radiodurans*. Transformation with two plasmids, pCasG (encoding the Cas effector) and pTarget (encoding the crRNA and homologous recombination template), enabled deletion of the carotenoid biosynthesis gene *crtB*. This knockout converted the characteristic pink colonies to white, providing a clear phenotypic marker (Chen et al., 2024). Subsequent plasmid curing on non-selective TGY medium yielded a markerless strain.

Chen et al. (2024) also compared several Class 2 effectors: LbCas12a (from *Lachnospiraceae*), FnCas12a (from *Francisella novicida*), AsCas12a (from *Acidaminococcus*), and SpCas9 (from *Streptococcus pyogenes*). LbCas12a and FnCas12a achieved the highest knockout efficiencies (~60% white colonies), while AsCas12a and SpCas9 were less effective (~40% and ~10%, respectively). These findings suggest that Cas12a nucleases outperform Cas9 in *Deinococcus*, highlighting the importance of effector choice for genome editing in extremophiles.

Beyond editing, CRISPR interference (CRISPRi) has also been explored in *D. radiodurans*, adding to its repertoire of established CRISPR/Cas tools (Table 2). Misra et al. (2023) engineered a type I E system (pCRD2) containing crRNA and the Cascade complex (Cse1, Cse2, Cas7, Cas5, Cas6e), but lacking Cas1, Cas2, and Cas3 to prevent DNA cleavage. Guided by 90–98 bp crRNAs, the complex bound downstream of the *phoN* start codon, sterically blocking transcription. This reduced *phoN* expression to ~10% of baseline activity and simultaneously suppressed *ssb* expression, demonstrating multiplexed knockdown capability. A type II-A dCas9 system was also tested, but poor transformation efficiency and suspected Cas9 toxicity (likely due to off target binding) limited its utility (Misra et al., 2023; Rostain et al., 2023).

**Table 2:** CRISPR/Cas systems for gene editing and interference in *Deinococcus radiodurans*.

Method	CRISPR/Cas system	References
CRISPR interference	Class 2, CRISPR-dCas9	Misra et al. (2023)
	Class 1, CRISPR-Cse1, Cse2, Cas7, Cas5, Cas6e	Misra et al. (2023)
Editing	Class 2, CRISPR-Cas9	Chen et al. (2024)
	Class 2, CRISPR-Cpf1	Chen et al. (2024)

Most recently, Yang et al. (2025) identified TnpB in *D. radiodurans* as a putative ancestor of modern CRISPR systems, closely related to Cas12f. TnpB functions as an RNA-guided endonuclease encoded within the genome, offering a compact alternative to full CRISPR machinery. Its potential for precise deletions, insertions, and gene silencing warrants further study as a next-generation editing tool.

CRISPR-based knockout and knockdown systems are particularly valuable for environmental engineering, where competing metabolic pathways can limit pollutant degradation or biofuel synthesis. For example, in *D. radiodurans*, both the pinene and deinoxanthin pathways compete for geranyl pyrophosphate (GPP). Disruption of *dr0862* (encoding phytoene synthase, a key enzyme in deinoxanthin biosynthesis) increased pinene yields, illustrating how targeted gene inactivation can redirect metabolic flux toward desired products (Helalat et al., 2021). Such strategies illustrate the promise of CRISPR in optimizing extremophiles for industrial bioremediation and sustainable fuel production.

## 5 Future outlook

### 5.1 Biosafety and ecological perspectives

*Deinococcus* poses minimal direct health risks to humans or animals, making pathogenic infection an unlikely concern; its notoriety stems from extraordinary stress resistance rather than virulence (Slade and Radman, 2011; Liu et al., 2023). This shifts the focus to ecological impact, a distinction that shapes the regulatory and operational safeguards required for environmental applications.

#### 5.1.1 Genetic and containment considerations

Laboratory strains of *Deinococcus* often carry antibiotic-resistance markers or metabolic plasmids for selection and monitoring. These should be removed or replaced with non-antibiotic systems prior to environmental release so as to prevent unintended spread (Aminov, 2011). A central concern is genetic persistence and the possibility of horizontal gene transfer (HGT), particularly via broad-host-range plasmids, which could transfer stress-tolerance traits such as solvent and metal resistance to native microbes (Popowska and Krawczyk-Balska, 2013; Macedo et al., 2022).

The polyploidy and exceptional DNA-repair capacity of *Deinococcus* present a double-edged sword; both traits enhance remediation performance under stress, yet they may also prolong environmental persistence if organisms are not adequately contained (Slade and Radman, 2011; Daly, 2023). Layered biocontainment is therefore essential, particularly given the absence of *Deinococcus*-specific containment systems to date (Brumwell et al., 2023). Recommended safeguards include engineered auxotrophy, genetic kill-switches, and physical containment; strategies that have been demonstrated in *Pseudomonas putida* and cyanobacteria, respectively, (Asin-Garcia et al., 2022; Asin-Garcia et al., 2024; Song et al., 2026), and through robust multi-layered CRISPR-based systems in other engineered organisms (Hayashi et al., 2024). Frameworks for evaluating and translating such measures to environmental deployment have been described recently (Rottinghaus et al., 2022; George et al., 2024).

#### 5.1.2 Environmental release concerns

Introducing any microorganism into the environment warrants rigorous ecological risk assessment. For *Deinococcus*, key considerations include persistence, ecological effects, genetic transfer, and contaminant rebound.

##### 5.1.2.1 Survival and spread

Engineered strains should ideally not persist after remediation or establish permanent populations beyond the treatment zone. Because *Deinococcus* is optimized for stress survival rather than competition under benign conditions, introduced strains are typically outcompeted by resident microbiota unless they retain a selective advantage (Thompson et al., 2005). Nonetheless, if the release site remains harsh (low nutrients, residual radiation), *Deinococcus* could expand beyond the intended footprint and modestly shift nutrient cycling by competing for organic matter (Thompson et al., 2005; Slade and Radman, 2011).

##### 5.1.2.2 Non-target effects on soil and water ecology

Microbes drive nutrient cycling, organic matter decomposition, and plant–microbe interactions. Introducing a new organism may therefore alter community composition and function. Field trials should monitor key ecological parameters such as nitrogen cycling rates, plant performance, and overall microbial diversity. When engineered *Deinococcus* carries metal resistance or sequestration functions, changes in metal speciation should be tracked to avoid unintended impacts on native metabolisms (Gao et al., 2022).

##### 5.1.2.3 Horizontal gene transfer and genetic biocontainment

Although cross-genus HGT in soil and water is generally rare, it does occur (Sun et al., 2019; Macedo et al., 2022). Regulators therefore require assurance that released organisms are free of antibiotic-resistance, virulence, or other hazardous traits, and recommend incorporating genetic safeguards such as kill-switches or synthetic auxotrophies (Rottinghaus et al., 2022; Hayashi et al., 2024).

##### 5.1.2.4 Containment rebound and biomass disposal

While *Deinococcus* can transform contaminants into more stable forms (e.g., converting soluble uranium into insoluble mineral precipitates), cell death and lysis may release previously adsorbed substances. Consequently, remediation plans must include recovery and compliant disposal of spent biomass to prevent secondary contamination (Manobala et al., 2019, 2021).

##### 5.1.2.5 Current risk assessment

No environmental harm has been attributed to naturally occurring *Deinococcus* species. The genus is recognized for its extreme stress tolerance rather than pathogenicity (Slade and Radman, 2011; Liu et al., 2023). Introduced strains commonly decline once selective pressures are removed. Bioaugmented bacteria rarely persist without a sustained advantage (Thompson et al., 2005). Nevertheless, direct field data for *Deinococcus* remain limited, so conservative safeguards are warranted (Thompson et al., 2005; Gao et al., 2022).

### 5.2 Challenges and limitations

#### 5.2.1 Fermentation and biomass production

Among extremophiles, *D. radiodurans* grows relatively slowly, with a doubling time of 2.6–4 h in optimized defined media. This contrasts sharply with laboratory *E. coli*, which doubles in about 20 min in rich media and typically reaches ~10^8–10^9 CFU mL^−1^ under standard conditions (Holland et al., 2006; Tuttle et al., 2021). By comparison, high-cell-density *E. coli* fermentations routinely exceed 10 g DCW L^−1^ (≈10^11–10^12 cells mL^−1^) and can achieve ≥60–100 + g DCW L^−1^ in fed-batch processes (Knorre et al., 1991; Riesenberg, 1991; Lee, 1996).

To overcome these growth limitations, industrial scale-up will likely require fed batch or continuous systems with cell retention, supported by media and buffer optimization at scale (He, 2009). Immobilization approaches such as biofilm reactors are particularly promising because they enable cell retention and prolonged operation without repeated inoculation (Armanu et al., 2025; Jhariya et al., 2025). Unlike conventional fermenters that rely on increasing planktonic cell numbers, biofilm reactors concentrate cells on surfaces, creating high local biomass density and improving contaminant contact. This means greater remediation efficiency per cell rather than simply producing more cells, which is especially advantageous for slow-growing organisms like *Deinococcus*. These advantages are particularly relevant for *Deinococcus* species, which exhibit natural traits conducive to biofilm formation and have already demonstrated practical success in engineered systems.

Natural surface-attachment capabilities and engineered biofilm systems illustrate the potential of this approach. Species within the genus *Deinococcus* exhibit traits conducive to biofilm formation, and these have been leveraged in practical applications. For example, *D. geothermalis* forms robust structures such as type IV pili and adhesion threads, while engineered *D. radiodurans* biofilms have demonstrated efficient uranium removal through column adsorption (Saarimaa et al., 2006; Manobala et al., 2019, 2021). By stabilizing cells under stress, biofilms support extended operation and reduce maintenance requirements, making them suitable for long-term remediation processes (Ogundolie et al., 2024). Immobilization strategies, including biofilm-based systems, have also proven effective for heavy metal removal and wastewater treatment, reinforcing their potential for scalable environmental applications (Saravanan et al., 2023).

For economic viability, low-cost substrates are preferred for fermentation. Several *Deinococcus* spp. metabolize common sugars. For example, *D. geothermalis* encodes an active xylanase capable of breaking down hemicellulose, supporting potential applications that utilize cellulosic hydrolysates or agricultural residues (Wang et al., 2024). Waste glycerol can serve as a carbon source for engineered *D. radiodurans*, underscoring the organism’s metabolic flexibility (Helalat et al., 2021). Medium optimization and targeted engineering to reduce the maintenance burden associated with stress-defense systems may further improve yields (Slade and Radman, 2011).

Capital and operating costs represent significant barriers to scale-up. Pilot-scale aerobic fermenters and associated utilities, including aeration, mixing and temperature control, are major cost drivers and slow growth extends run times and labor. While the robustness of *Deinococcus* may ultimately enable relaxed sterility, potentially through the use of oxidative or radiation pulses, this requires validation at scale (Saravanan et al., 2023; Koolivand et al., 2024).

#### 5.2.2 Formulation, transportation, and deployment

After cultivation, *Deinococcus* biomass must be safely transported to remediation sites. *D. radiodurans* is unusually stable in dry form and tolerates prolonged desiccation, enabling shipment as dried powders or lyophilized pellets for on-site rehydration. Trehalose and related compatible solutes associated with desiccation tolerance can be incorporated into protective matrices to further improve stability (Slade and Radman, 2011; Farfan Pajuelo et al., 2023). Immobilizing cells in alginate beads or on inert carriers (e.g., biochar) can facilitate handling and controlled release (Wu et al., 2022; Ogundolie et al., 2024; Schommer et al., 2024). Deployment options such as soil mixing, groundwater injection, or closed-loop bioreactors each present logistical constraints as well as permitting needs (Gao et al., 2022; Koolivand et al., 2024).

#### 5.2.3 Economic feasibility

The economic feasibility of *Deinococcus*-based bioremediation at commercial scale remains unproven, with viability depending on biomass production costs, removal efficiency, and waste-handling requirements relative to chemical treatment or monitored natural attenuation. Although no full-scale deployment has been reported, laboratory studies across contaminant classes consistently show high removal efficiencies. For example, *D. radiodurans* biofilms (DR1-bf^+^) cultivated with 20 mM Ca^2+^ removed ~75 ± 2% of U(VI) from a 1,000 mg/L uranyl nitrate solution within 30 min (Manobala et al., 2019, 2021; see also Section 3.1). Findings like this provide a clear technical basis for advancing to pilot-scale testing.

### 5.3 Regulatory considerations in the United States

Environmental use of live microbes in the U.S. is governed by the Coordinated Framework for the Regulation of Biotechnology, which defines the roles of the EPA, FDA, and USDA (EPA, 2017; USDA, 2017; About the Coordinated Framework | Animal and Plant Health Inspection Service, n.d.).

#### 5.3.1 EPA oversight

The EPA regulates “new” (intergeneric) genetically engineered microorganisms under the Toxic Substances Control Act (TSCA) Section 5, typically requiring a Premanufacture Notice (PMN). This review evaluates factors such as toxicity and pathogenicity, environmental fate and persistence, gene transfer potential, containment measures, and survival (EPA, 2015a, 2015c). Naturally occurring *Deinococcus* strains without intergeneric DNA may be exempt, but engineered strains undergo TSCA Section 5 review and may be subject to use restrictions or mandatory monitoring requirements.

#### 5.3.2 NEPA review

Projects on federal land or those receiving federal funding may require an Environmental Assessment (EA) or a more comprehensive Environmental Impact Statement (EIS), depending on the scope and potential impacts of the proposed project (NEPA, 2009; EPA, 2013).

#### 5.3.3 Waste management

Disposal of spent *Deinococcus* biomass or secondary wastes must comply with the Resource Conservation and Recovery Act (RCRA) hazardous-waste requirements (EPA, 2018, 2024). For radiological sites, DOE Order 458.1 (Radiation Protection of the Public and the Environment) and NRC low-level waste guidance apply (NRC, 2016; DOE, 2011).

#### 5.3.4 Groundwater injection

Subsurface injection approaches may require an Underground Injection Control (UIC) permit under the Safe Drinking Water Act (EPA, 2015b). State agencies with primacy may also impose additional permitting requirements.

## 6 Conclusion

*Deinococcus* offers an intriguing biological tool for addressing some of the toughest cleanup challenges. These organisms can survive where other microbes fail and, once equipped with the appropriate genetic pathways, can act on contaminants under the harshest of environmental conditions. Laboratory studies have convincingly shown that *D. radiodurans* and related species can accumulate radionuclides and metals and degrade organic solvents even under severe irradiation challenge. These capabilities position *Deinococcus* as a unique candidate for remediation at the interface of radiation and pollution.

Nevertheless, translating *Deinococcus*-based remediation into field reality remains non-trivial. At present, the lack of quantitative, comparative performance data and the lack of pilot or full scale trials limits firm conclusions about the relative efficiency or cost effectiveness of *Deinococcus* or the practical advantages of its deployment over established remediation techniques. Furthermore, technical hurdles span the entire deployment chain including fermentation optimization for yield and cost, formulation of cells for stability and transport, and integration with engineering systems such as bioreactors or immobilized cartridges. Economic feasibility will ultimately depend on site-specific factors and the value placed on avoiding more disruptive cleanup approaches.

Safety and regulatory compliance are equally critical. Although *Deinococcus* itself is non-pathogenic, engineered strains and mobilized pollutants require careful monitoring and control. Risk mitigation strategies, such as encapsulation, immobilization, and genetic safeguards (e.g., kill switches), should be incorporated into project plans from the outset. Regulatory approval pathways (EPA/TSCA in the U.S.; GMO and biocidal directives in the EU) demand substantial data, making early collaboration with regulators essential.

Encouragingly, advances in synthetic biology and genomics provide strong grounds for optimism. Programmable genetic circuits could enable *Deinococcus* to sense pollutant levels and self-terminate once thresholds are met, while expanded genomic insights may uncover additional native catabolic enzymes across diverse species. Overcoming technical, logistical, and policy barriers will require a coordinated multidisciplinary approach spanning microbiology, process engineering, economics, and regulatory science.

In conclusion, the resilience and metabolic adaptability of *Deinococcus* make it a compelling candidate for bioremediation. While challenges remain, emerging tools in synthetic biology and genetic engineering offer a clear path forward. This review has outlined the current state of knowledge in the field and identified key gaps. By addressing knowledge gaps with practical, scalable solutions, *Deinococcus*-based strategies can progress from laboratory promise to real-world application, providing safe, effective options for remediating some of the planet’s most persistent contaminants.
